# Landscape Dynamics Improved Recreation Service of the Three Gorges Reservoir Area, China

**DOI:** 10.3390/ijerph18168356

**Published:** 2021-08-06

**Authors:** Dengyue Zhao, Mingzhu Xiao, Chunbo Huang, Yuan Liang, Ziyue An

**Affiliations:** 1 Research Center for Spatial Planning and Human-Environment System Simulation, School of Geography and Information Engineering, China University of Geosciences, Wuhan 430078, China; zhaodengyue@cug.edu.cn (D.Z.); anziyue0526@cug.edu.cn (Z.A.); 2 School of Arts and Communication, China University of Geosciences, Wuhan 430074, China; xiao_mingzhu@cug.edu.cn (M.X.); liangyuan612@cug.edu.cn (Y.L.); 3 Hubei Engineering Technology Research Center for Forestry Information, College of Horticulture and Forestry Sciences, Huazhong Agricultural University, Wuhan 430070, China

**Keywords:** cultural service, ecological model, landscape visits, spatial heterogeneity, landscape planning

## Abstract

Spatio-temporal variations of recreation service not only could help to understand the impact of cultural services on human well-being but also provides theoretical and technical support for regional landscape management. However, previous studies have avoided deeply quantifying and analyzing it or have simply focused on assessing recreational service at a single period in time. In this study, we used the InVEST model to evaluate the spatio-temporal variations of recreation service in the Three Gorges Reservoir Area and demonstrated the impact of recreation service on landscape dynamics. The results demonstrated that recreation service increased significantly and presented a significant spatial heterogeneity. Although afforestation and urban expansion both could significantly increase recreation service, the recreation service proxy of the non-vegetation landscape is far higher than that of the vegetation landscape. This finding indicated that human landscape is more attractive to tourists than the natural landscape, so we recommend to strengthen the infrastructure construction for enhancing the accessibility of natural landscapes. Moreover, we propose other constructive suggestions and landscape-design solutions for promoting recreation service. This study shifted the static environmental health assessment to the analysis of recreation service dynamics, bridging the regulatory mechanisms of ecosystem services involved in cultural services.

## 1. Introduction

Ecosystem services are the benefits that people obtain from ecosystems [1,2]. The Millennium Ecosystem Assessment classified ecosystem services into four sections, i.e., provisioning service, regulating service, supporting service, and cultural service. Cultural services are defined as non-material benefits that people obtain from the ecosystems through spiritual enrichment, cognitive improvement, reflection, recreation, and aesthetic experience [3]. Cultural services are often generated by the interdependence of other key ecosystem services [4]. Many scholars have already studied some relevant ecosystem processes and services related to cultural services [5,6,7,8]. For example, Dickinson et al. [9] conducted a qualitative review of literature to identify key characteristics of cultural ecosystem services and challenges to cultural ecosystem services research. Dou et al. [10] analyzed the impact of the Chinese ecological restoration policy on the cultural ecosystem services of rural communities by taking Guyuan city in China’s Ningxia Hui autonomous region as a case study. It makes the role of cultural services in social-natural ecosystems increasingly recognized and understood by people. However, it is difficult to estimate and value because of the conceptual ambiguity, the interdisciplinarity, and the controversial research methods of cultural services [2,11]. Research on ecosystem services is dominated by the natural sciences and economics, but cultural services research requires social science and humanistic perspectives [12]. Scientists and policy makers need to consider the multidimensional relationships between human well-being, nature, as well as cultural and social values to capture the relevant benefits and understand the underlying mechanisms and drivers of change [13]. Integrating cultural services into an ecosystem services assessment and trade-off framework in the same way as regulation services still is a challenging issue. Moreover, numerous studies neglect cultural ecosystem services, which were consistently weaker in terms of assessment and trade-offs [14,15,16]. But the interdisciplinarity of cultural services determines its enormous research potential and significance. It also is an important basis for integrating ecosystem services to trade-off, connect, and solve scientific problems in multiple fields [11]. Therefore, it is necessary to evaluate cultural services to improve our understanding of the interactions between society and ecosystems.

Since quantifying and analyzing cultural services is difficult, previous studies have avoided and neglected these services [6,17,18]. The assessment indicator of cultural services remains imperfect; therefore, many scholars have used a variety of methods to better understand and quantify cultural services [14,19]. Richards et al. [20] analyzed social media photos with the Google Cloud Vision image-recognition algorithm to evaluate the cultural ecosystem services in Singapore. Thiele et al. [21] applied an indicator framework for spatially assessing the cultural ecosystem services provided in German river landscapes. Besides, the monetized accounting and quantitative scoring methods are also the main measures for assessing cultural service. However, monetized accounting is subject to numerous controversies in terms of assessment errors and theoretical foundations, while quantitative scoring is accepted by more researchers [6,14]. In addition, the benefits that cultural services enhance for humanity are not tangible and therefore are difficult to assign a monetary value [22]. Moreover, many studies have mostly conducted static studies with survey or assessment results of a single period, while dynamic studies monitoring the spatial and temporal variations of regional cultural services are still lacking. Therefore, there is an urgent need to dynamically identify and observe the impact of cultural services on human well-being. The spatial-temporal evolution of cultural services is usually characterized by GIS tools [19], selecting specific cultural service types, such as recreation proxy [14], ecotourism proxy [23], cultural heritage [24], and educational value [19,25], to map the spatial distribution. Meanwhile, the past dynamics and the future changing trends of these service indicators should be analyzed and predicted to document the temporal evolution of cultural services. This approach not only helps people understand the impact of cultural services on human well-being but also provides theoretical and technical support for regional planning and land-use policies.

The Three Gorges Reservoir Area (TGRA) is rich in natural and humanistic tourism resources, and tourism benefits are an important part of the region’s economy [26,27,28]. There are 177 A-class scenic spots, including 13 5A-class scenic spots, such as Dazu Rock Carvings, Three Gorges Dam, and Badong Shinlong Stream [28]. But except for the Yangtze River Three Gorges coastal and the Daning River Small Three Gorges, which have high visibility and attraction, the tourist sites in the hinterland of the reservoir area are not very influential [29]. In recent years, the level of the tourism economy in the TGRA has gradually improved, but there are still many problems. For example, there are conflicts between regional development and ecological protection [30], and the development of ecological tourism resources in the reservoir area is weak. Tourism infrastructure (e.g., road network density and grade) and hospitality facilities (e.g., hotels) are lagging. Therefore, it is necessary to vigorously advocate ecological tourism in the reservoir area and strengthen infrastructure construction to improve the utilization rate of natural and humanistic tourism resources and promote the economic development of the TGRA [31]. However, with the impoundment of the Three Gorges Dam and the increase in water level in the reservoir area, some humanistic landscapes, such as Zhang Fei Temple, have been submerged. Although other landscapes, such as the Baidi City in Fengjie, have not been submerged, it has been turned into an isolated island. To effectively save tourism resources, people have preserved some humanistic landscapes by relocating them. However, after the construction of the Three Gorges Water Conservancy Project, water storage has brought new tourism resources, such as the High Gorge Lake, the Three Gorges Dam, and water activities like paragliding. Reservoir storage has a profound impact on tourism development in the TGRA. It not only destroys some original landscapes but also brings humanistic tourism resources to these new landscapes. Therefore, the TGRA accomplished a transition of the humanistic tourism resources from the “Old Three Gorges” to the “New Three Gorges” [32]. The transformation alters the structure and function of tourism products in the reservoir area [33]. Therefore, analyzing the evolution of cultural services in the TGRA in the past decades is important for balancing regional economic development and ecological conservation.

In this study, we used the InVEST model to evaluate the recreation service of the TGRA and analyze its spatial and temporal variations. A shift from assessing the static recreation service to analyzing the recreation service was conducted to explore the feedback principle of cultural services on the landscape configuration evolution and to attempt to bridge the regulatory mechanisms of ecosystem services involved in cultural services. The specific objectives are as follows: (1) constructing a multiple regression model for assessing the recreation service; (2) characterizing the spatial and temporal variations of the recreation service in the TGRA; and (3) demonstrating the differences of recreational services for various landscapes and the response of recreational service on the landscape dynamics to provide a theoretical basis for the improvement of recreation services in the TGRA.

## 2. Materials and Methods

### 2.1. The Study Area

**Figure 1:**
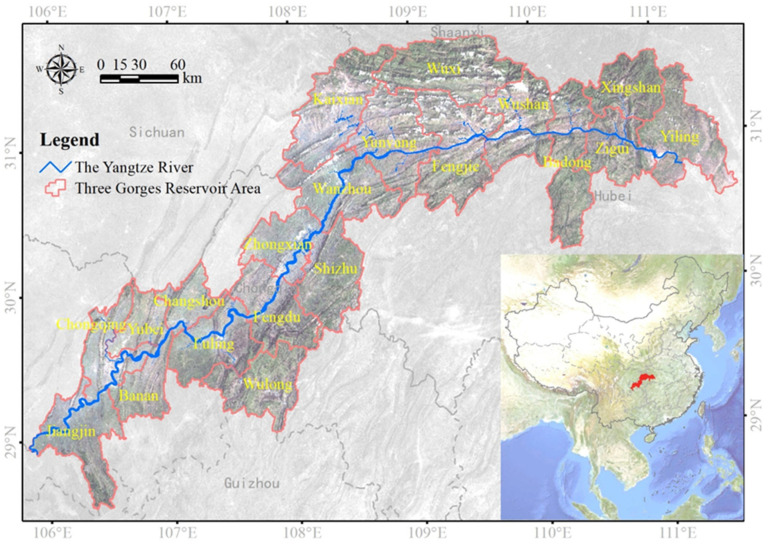
Location of the Three Gorges Reservoir Area (TGRA), China.

The TGRA is located in the upper and middle reaches of the Yangtze River (Figure 1) and lies between 28°31′–31°44′ N and 105°50′–111°40′ E. The reservoir area is located in the Sichuan Basin and the Yangtze River Basin, which joins the plain rivers. Its easternmost part is Yichang, Hubei Province, and its westernmost part is Baxian County, Chongqing City. It is about 5.8 × 104 km^2^, containing four counties in southwestern Hubei (Yiling, Zigui, Xingshan, and Badong) and 16 districts and counties in east-central Chongqing (Wuxi, Wushan, Fengjie, Yunyang, Kaixian, Wanxian, Zhongxian, Shizhu, Fengdu, Wulong, Fuling, Changshou, Yubei District, Banan District, the main city of Chongqing, and Jiangjin City). In 2018, the resident population of the TGRA was 21,025,900, with an urbanization rate of 64.42%. The TGRA has a humid, mid-subtropical monsoon climate, with a mean annual temperature of 20 °C and mean annual precipitation of 1200 mm. It is a typical biodiversity hotspot with rich plant resources in China.

The natural resources mainly include rapids, canyons, caves, etc. There are also unique human resources, such as stone carvings, hanging coffins, and ancient architectural complexes. The natural landscapes that are generated by the special topography and transitional climate of the TGRA mainly include mountain and water landscapes. The reservoir area includes more than 10 main tributaries, such as the Daning River, Wu River, and Jialing River, presenting a variety of water landscapes. The mountain landscapes include the twelve peaks of Wu Mountain, such as Wind Box Gorge, Goddess Peak, Bawu Gorge, Jingyinkuijia Gorge, and Cuokai Gorge, as well as the Little Three Gorges, Xiniuwangyue Gorge, and Niuganmafei Gorge in the Daning River Basin. The cultural landscapes contain Zhang Fei Temple, Luyou Cave, White Crane Ridge, Eight Formations, Zhanghuanhou Temple, Du Fu Thatched Cottage, Han Dynasty Stone Que, Baojian Gorge, etc. Based on this special natural geographical environment, tourism in the reservoir area has been in a steady growth stage. In 2018, the region received 564,096,800 tourists, accounting for 21% of the total number of tourists received nationwide, and generated a total tourism revenue of $39.58 billion, accounting for 21% of the total tourism revenue [28]. However, the development level of each county is still unevenly affected by many factors, so the research still needs to be further deepened [34].

### 2.2. Ecological Model of Recreation Service

In this study, the InVEST Recreation model was used to assess the recreation service of the TGRA. To quantify the natural environment and socio-economic values, the model predicts visits to the landscape based on its location, accessibility, and the infrastructure that attracts people to choose recreational locations [35,36]. Due to the lack of historical data on landscape visits, the geotagged images posted on the Flickr website (https://www.flickr.com/, accessed on 13 November 2018) were used as a proxy for the number of visits to predict the spread of person-days of recreation in space. Flickr is a popular SM platform for nature photographers, with over 90 million monthly active users in 2018, allowing users to access publicly available content for non-commercial use through their Application Programming Interface (API) [37]. Using the location information on photos as well as the photographer’s username and the date that the photo was taken, the InVEST model can calculate the total photo-user-days for each grid cell or polygon. The average annual number of photo-user-days is then returned to the user. Next, the model evaluates the contribution of each attribute to the number of visits through a multiple linear regression equation. Additionally, the spatial distribution of visits is estimated based on the regression equation (Equation (1)).

(1) $$y=a_{0}+a_{1}\times x_{1}+a_{2}\times x_{2}+\cdots +a_{p}\times x_{p}$$

where *y* is the number of visits, and *x* is the independent variable associated with the number of visits. *a* is the parameter of the independent variable.

The predictor variable in this algorithm is the annual number of photo visits, while the independent variables mainly include natural resources, such as forests and lakes; infrastructures, such as roads and hotels; and variables related to the cost of visits, such as the range of activities associated with the distance from the airport [35,38].

### 2.3. Assessing the Recreation Service

Many ecosystems have important value as places where people can come for rest, relaxation, refreshment, and recreation [2]. With the increase in population, wealth, and leisure time, the demand for recreation in natural areas and man-made landscapes will likely continue to grow in the future. According to the InVEST model user’s guide, a major and growing component of recreation is its nature orientation, which includes interaction with the natural environment. Additionally, some recreational activities depend on other attributes, such as infrastructure and cultural attractions. Therefore, the recreation service is influenced by both natural environmental and socio-economic conditions [5,7,19]. The natural environmental conditions include topography, hydrological environment, and surface landscape type. The socio-economic conditions also include the distribution of tourist attractions, accessibility, and accommodation conditions.

We selected 13 indicators (nine natural environmental factors and four socio-economic factors) from the two systems of natural environmental conditions and socio-economic conditions based on the collection of the natural environment and socio-economic data in the TGRA (Table 1). Temperature and rainfall data from natural and environmental factors were provided by the Data Center for Resources and Environmental Sciences, Chinese Academy of Sciences (http://www.resdc.cn, accessed on 13 November 2018). At the same time, we downloaded the 30 m resolution DEM data (ASTER Global Digital Elevation Model V002, http://www.gscloud.cn/, accessed on 13 November 2018) to obtain the elevation and used ArcGIS spatial-analysis tools (Version 10.2.2) to generate the slope map. Other natural and environmental factors, such as SOC and land-use data, were provided from other studies [30,39]. Moreover, the road-density data was provided by the National Geomatics Center of China (NGCC) (http://ngcc.sbsm.gov.cn/, accessed on 13 November 2018), while the other three socio-economic factors were acquired from Baidu maps.

### 2.4. Statistic Analysis

#### 2.4.1. Trend Analysis

Trend analysis was used to identify the changing trend of recreation services proxy. Many different statistical methods (such as correlation and regression analyses, time-series analyses, and methods based on nonparametric statistics) are available for detecting and estimating possible trends in the environmental variables of interest. Referring to the relevant studies [40], we chose the least squares linear regression model (Equation (2)) to analyze and describe the changing trend of recreational services in the TGRA. In this paper, we obtained a sample size of 615, and the freedom of the regression model was 607. Besides, the multiple R-squared is 0.7452, while the adjusted R-squared is 0.7418. The statistical significances of these variations were reflected by the *t*-test and *p*-value of the modeled slope.

*y* = *ax* + *b* (2)

where *y* is the recreation service indicator, and *x* is the time (year). *a* is the modeled slope, which could reflect the annual variation rate of the recreation service indicators, and *b* is the intercept of the regression model.

#### 2.4.2. Correspondence Analysis

Correspondence analysis is a data analysis model that attempts to consider the distance between study objects by combining classification methods with graphical modeling [31,41,42]. This method was employed to identify the relationship between the landscape changes and the recreation services changes. Based on their spatial information, we counted the grid number of the various characteristics of cultural services at a 1-km grid scale. In applying correspondence analysis, the number of grids was discretized to place the data into a contingency table. The column names contain the two pollutant change characteristics and their names, while row names contain the names of the landscape-change types. With the help of the correspondence analysis function in R language, the relationship between landscape-change characteristics and recreation-service-change characteristics was explored using the distances between row and column categories, which were embedded in the corresponding analysis plots.

## 3. Results

### 3.1. Recreation Services of the TGRA

We introduced into the optimal regression equation with eight independent variables, of which four were natural environmental factors and four socio-economic factors, respectively (Table 2). All independent variables passed the *t*-test at 95% significant level, with the density of tourist attractions and the proportion of water bodies passing the *t*-test at 99% significant level. Moreover, the adjusted R^2^ of this regression model was approximately 0.75, indicating that the reliability of this regression model is strong, and this model can meet the needs of our study.

The results of multiple regression analysis demonstrated that the recreation service of the TGRA was positively correlated with the density of tourist attractions for which the estimated parameter was 0.44. Meanwhile, the recreation service was also positively correlated with the other four variables (including minimum distance to hotel, road density, forest coverage, and proportion of water area). Additionally, their estimated parameters in the regression model were 0.55, 7.77, 0.34, and 1.69, respectively. However, the recreation service of the TGRA was negatively correlated with the minimum distance to traffic station, elevation, and proportion of built-up area. Their estimated parameters were −0.04, −0.01, and −0.65, respectively.

Based on the optimal regression model of recreation service (Figure S1), the recreation service proxy of the TGRA increased from 5.23 in 2000 to 10.76 in 2015. The results of the trend analysis demonstrated that the recreation-service proxy of the TGRA was growing at a linear rate of 0.37 per year. Within each period, the increased rate of the recreation service proxy was lower during 2000–2005, while the higher growth rate was observed during 2010–2015.

### 3.2. Spatial and Temporal Variations of the Recreation Service

**Figure 2:**
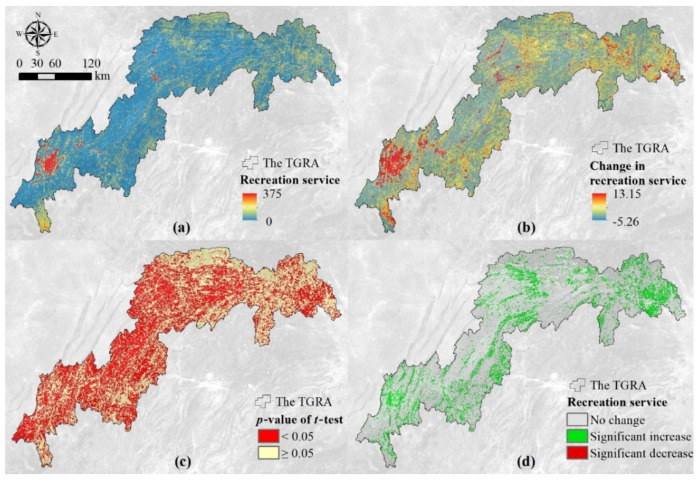
Spatial variation of the recreation-service proxy in the TGRA between 2000 and 2015. Average (**a**) and the modeled slope (**b**) of the recreation service, *p*-value of *t*-test for the slope (**c**), and the changes of recreation service (**d**). Note: In the figure (**d**), “No change” shows that the recreation service did not significantly change (slope = 0), and “Significant increase” refers to slope > 0 and *p* < 0.05, while significant decrease refers to slope < 0 and *p* < 0.05.

Grids with a high recreation service were mainly distributed around the built-up areas in the TGRA, and the recreation-service proxy was up to 375 (Figure 2a). In addition, the recreation service was relatively higher in the northeastern and southwestern regions of the reservoir area. According to the trend analysis (Figure 2b), the maximum growth rate of the recreation-services proxy was as high as 13.15 per year and was mainly located near construction sites and the Yangtze River. The results of the *t*-test for the change rate of the recreation-services proxy (Figure 2c) showed that about 50% of the grids failed to pass the *t*-test at the 95% significant level. The grids with a significant increase in recreation services accounted for 19% of the study area and were mainly distributed in blocks along the banks of the Yangtze River. In addition, the recreation service did not significantly change in 30.91% of the TGRA. The grids with a significant decrease in recreation services accounted for only 0.04% of the TGRA (Figure 2d).

**Figure 3:**
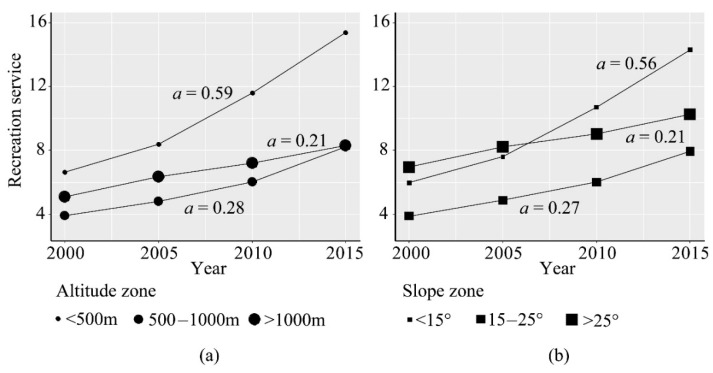
Temporal variations of the recreation-service proxy in different zones of altitude (**a**) and slope (**b**) in the TGRA. Note: We used a least square linear regression model to fit the recreation proxy. The changing trend is described by the modeled slope, which is *a* in the figure.

The recreation service vertical characteristics exhibited a distinct spatial heterogeneity in the TGRA (Figure 3). In the altitude hierarchy (Figure 3a), the recreation service was highest in the <500 m zone, where it had the largest growth rate, with 0.59 per year for the proxy. However, the recreation service was lowest in the 500–1000 m zone, where it had a smaller growth rate, with 0.28 per year for the proxy. The two zones showed exponential growth in the recreational service, while the >1000 m zone showed linear growth, with a rate of 0.21 per year for the proxy. In the slope gradation (Figure 3b), the recreation service was lower in the <15° zone than those in the >25° zone in 2000 and 2005. However, the recreation service significantly increased dramatically after 2005 in the <15° zone, and this zone turned into the largest slope subdivision for recreation services. In addition, the growth rate of the recreation-service proxy was highest in the <15° zone, with a value of 0.56 per year. The recreational service was at its minimum in the 15–25° zone, where the growth rate was 0.27 per year for the proxy. Moreover, the recreation-service proxy in the 15–25° zone was always less than 8 between 2000 and 2015. Meanwhile, the recreation service proxy in the >25° zone had the smallest growth rate of 0.21 per year.

### 3.3. Spatial Heterogeneity of the Recreation Service

We analyzed the spatial heterogeneity of the recreation service in the 20 counties (Figure S2a). The recreation service was highest in Chongqing, where the proxy was about 30.94. Meanwhile, the recreation service was lowest in Zhongxian, where the proxy was only 1.76. The recreation service was higher in these three counties, which are Yiling, Yubei, and Chongqing. Their recreation-service proxies exceeded 10. Additionally, the recreation-service proxy was less than 5 in the other six counties in the middle of the reservoir area, such as Kai County, Fengjie, and Zhongxian. According to the trend analysis, the modeled slope of the recreation-service proxy reflected the dynamic characteristics of recreation services in each county (Figure S2b). Meanwhile, the modeled slope passed the *t*-test in the 14 counties. These results indicated that recreation services had increased in all 20 counties. Among them, Chongqing and Yubei had higher growth rates, with values of 1.596 and 1.193, respectively. While the growth rates of Xingshan, Fengjie, and Zhongxian were lower, with values of 0.176, 0.149, and 0.143, respectively, only Fengjie passed the significance test among these three counties.

Our study conducted a hierarchical clustering analysis to characterize the recreation service for each county in the TGRA (Figure S3). The distribution and dynamic characteristics of the recreation service in the 20 counties within the reservoir area can be divided into two categories as follows: (1) counties with a high level of the recreation service— there are two counties, i.e., Yubei and Chongqing, which are distributed in the western part of the reservoir area. Their recreation service proxies are up to 30; and (2) counties with a low level of the recreation service, such as Zigui, Fengjie, and Zhongxian. Their recreation service proxies are less than 10 between 2000 and 2015.

### 3.4. Relationship between Landscape Dynamic and Recreation Service

The capacity of the recreation service altered among these land-use-change types, but all showed an increasing trend from 2000 to 2015 (Figure S4). The recreation-service proxy increased from 24.32 in 2000 to 42.87 in 2015 for the other land use, which includes built-up land and water bodies. Meanwhile, the growth rate of the proxy was higher, with a value of 1.26 per year. The recreation services of vegetation landscapes were lower, and the proxy was fluctuating consistently between 4 and 8 during 2000–2015. The recreation-service proxy of forestland increased from 5.56 in 2000 to 9.52 in 2015, and the growth rate of the proxy was 0.26 per year. At the same time, the growth rates of the proxies were 0.29 and 0.38 per year for deforestation and cropland, respectively, while grassland had the lowest growth rate of the recreation-service proxies, with a value of 0.08 per year.

**Figure 4:**
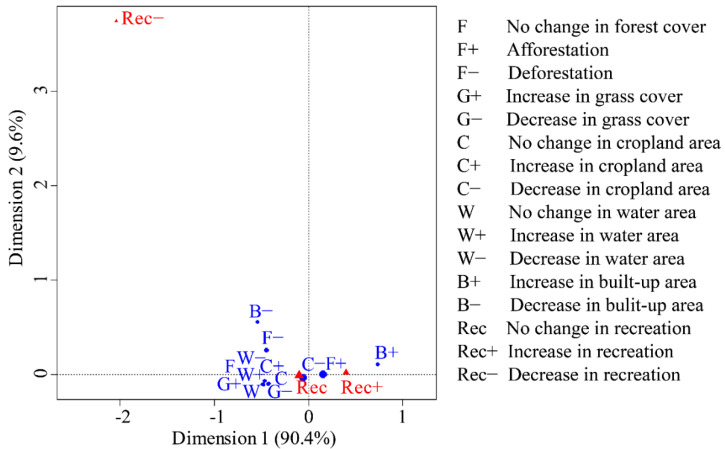
Correspondence analysis between recreation-service changes and land-use changes.

Correspondence analysis between landscape changes and recreation-service changes showed that the first dimension explained 90.4% of the variance, while the second dimension explained only 9.6% of the variance. According to the corresponding analysis diagram (Figure 4), most of the landscape-change categories were concentrated near the “no change in recreation” indicator (Rec). It is illustrated that these landscapes could not significantly alter the recreation service. In addition, the concentration also demonstrated that they have extremely similar impacts on the recreational service. The loss of afforestation, cropland, and the increase in construction land were distributed near the “increase in recreation” indicator (Rec+), showing that these landscape changes could effectively contribute to the enhancement of recreational services. Rec and Rec+ were close together but distant from the “decrease in recreation” (Rec−), and there were no categories of landscape change near Rec-. It indicated that the landscape evolution of the TGRA between 2000 and 2015 had not significantly decreased the recreational service.

## 4. Discussion

### 4.1. Assessing and Characterizing the Recreation Service

Leisure and tourism are important economic components in many countries and the tertiary sector, positively impacting the quality of life, habitat, social connections, physical well-being, and other intangibles in many ways [14]. Recognizing and valuing the benefits of ecosystems in supporting nature-based recreation and rapidly growing ecotourism can play an important role in alleviating poverty, especially in developing countries where much of the world’s natural capital is located [43]. Ferraro et al. [44] demonstrated that nearly two-thirds of poverty reduction can be attributed to ecotourism related to recreation services. To quantify this service, the natural ecological factors and human social factors were used to construct a multiple linear regression model based on the InVEST model for assessing the recreation service of the TGRA. We ran the model iteratively to select suitable independent variables. However, it is difficult to compare the relative contributions of the respective variables to the predictor variables because these independent variables are spatial data [35]. Overall, the adjusted R2 of the multiple linear regression model was as high as 0.75, indicating that the reliability of this regression model is strong, and the model could be used to assess the recreation service of the TGRA.

Based on the model of the recreation-service proxy, the recreation-service proxy of the TGRA presented a linear growth during 2000–2015 (Figure S1). The minimum growth rate of the recreation-service proxy occurred during 2000–2005, while the maximum growth rate occurred during 2010–2015. As the recreation service is affected by the interactions of the natural environments, infrastructures, and cultural identity, the larger growth rate in the later period might be caused by the improvement of ecological conditions in the reservoir area and the rising cultural demands of the people in this area [26,32]. The TGRA has unique natural landscape resources and superior human landscape resources, which can combine natural sceneries, cultural characteristics, socio-economic development, and water conservancy projects into a whole. This provides residents with a range of cultural services for leisure and recreation, such as experiencing nature, recreational activities, relaxation, outdoor sports, and aesthetic experiences. It contributes to the mental and physical recovery of residents. Therefore, there is a strong development demand for cultural services that has contributed to the increase of the recreation service in this area.

### 4.2. Spatial Heterogeneity of Recreation Services

The recreation service was higher in Yiling, Yubei, and Chongqing. In particular, the recreation-service proxy was more than 35 in Chongqing, far exceeding other regions (Figure S3). Although the natural resources of these three counties are far worse than that of other counties, their population density, attraction number, and infrastructure development level are far higher than that of other counties. This demonstrated that infrastructure development and construction have a strong impact on the recreation service. A similar finding was reported by Heagney et al. [44], who identified and established the infrastructure as the primary driving factor of the recreation demand. Moreover, grids with middle and high recreation-service levels in the TGRA were mainly concentrated in and around the built-up areas. The recreation service was higher in a few of the natural landscapes, but the proxy values were much lower than that of the cultural landscapes (Figure 2). Therefore, although the reservoir area has many excellent natural resources, the shortages of the infrastructure and tourism developments make the natural landscape more difficult to access than the cultural landscape. Moreover, the multiple regression model of the recreation-service proxy documented that the recreation service was positively correlated with the road density and the minimum distance to hotels (Table 2), which further illustrated the urgency of building infrastructure. Therefore, the development and construction of infrastructure are vital for addressing the travel and living issues of visitors.

According to the vertical characteristics of the recreation service in the TGRA (Figure 3), we found that human activities are most intense in the grids with a lower altitude and gentler slope. Meanwhile, there are more cultural landscapes in these grids. In contrast, although the natural landscapes are excellent in the regions with higher altitudes and steeper slopes, the landscape accessibility is lower due to the lack of infrastructure. On the one hand, the vertical characteristics of the recreation service indicated that the human landscape of the reservoir area is more attractive to tourists than the natural landscape. On the other hand, it demonstrated the incompleteness of infrastructure development and construction in these areas with higher altitudes and steeper slopes. Visitor demands in remote areas are associated with facilities, such as day-use areas, retail, and camping, reflecting the lack of infrastructure in the surrounding areas [44]. Therefore, it is necessary to strengthen the construction of infrastructure in the high-altitude areas with beautiful natural landscapes and enhance the accessibility of scenic areas to attract tourists and upgrade the recreation service in the region.

### 4.3. Response of Recreation Services to Landscape Evolution

The recreation services significantly varied among the vegetation landscapes and the non-vegetation landscapes (Figure S4). This might be due to the urbanization development and the infrastructure construction. In addition to causing significant environmental changes, urbanization leads to dramatic changes in people’s lifestyles [45]. The increase in people’s leisure time has also expanded the demand for leisure and entertainment. Additionally, the demand for a good quality of life and a healthy urban environment is also rising significantly [46]. By comparing the distance between landscape evolution types and the recreation-service-change types in the corresponding analysis (Figure 4), both afforestation and urban expansion could effectively increase the level of the recreation service. Jůza et al. [47] also found that forests are recreational places for all ages and have a high value for recreational services. In contrast, the recreation service had no significant changes in most of the other landscape changes. Besides, the landscape dynamics during the past 15 years have not directly decreased the potential of the recreation service. This may be because revegetation has increased the potential recreation service of the natural landscape, and urban expansion has strengthened the infrastructure development and construction, thus effectively increasing the potential recreation service of the human landscape [32]. Therefore, ecological protection and urban construction in the reservoir area both have enhanced the potential ability of the recreation service from different perspectives.

As the urbanization process accelerates, the suburban recreational forests have received more and more attention. The result was in line with Prete et al. [48], who confirmed the growing interest in forests, including the demand for forest tourism and recreational activities. In the forest ecosystem, people can be freed from artificial environments, sedentary lifestyles, and virtualized social relationships [49]. Besides, leisure and tourism in the forest landscape could help people reduce stress, stimulate physical activity, and promote social cohesion [49]. However, visitors prefer the inner city for daily activities, such as dog walking and jogging, over the forests around the city [45]. Therefore, a tourist attraction located in a densely populated area can attract more visitors due to its convenient location. This demonstrates that the level of urbanization construction and infrastructure development plays an important role in leisure and recreation. However, the multiple regression model indicated that the recreation service is negatively correlated with the proportion of built-up area (Table 2). The shortages of green infrastructure in cities may partly explain this phenomenon. Therefore, we suggest that policy makers should pay attention to the reasonable allocation of built-up land and scenic woodland when conducting urban planning so as to create an environment-friendly city.

### 4.4. Landscape-Planning Suggestions

The TGRA has both unique natural landscape resources and high-grade human landscape resources, which contributes to a region of high-quality tourism resources. But the high-intensity vegetation destruction in the past decades and the barren hills on both sides of the Yangtze River have seriously affected the scenery of the reservoir area [31]. If effective measures are not taken in time, it will not only cause the waste of tourism resources but will also be detrimental to economic development. As an important eco-economic zone, eco-tourism is an essential economic component of the reservoir area. Therefore, the rational regulation of scenic forests in the reservoir area and cities can improve the habitat conditions and promote construction and the development of tourism. We provided some landscape-planning schemes to attract more visitors and increase the cultural services of the TGRA.

**Figure 5:**
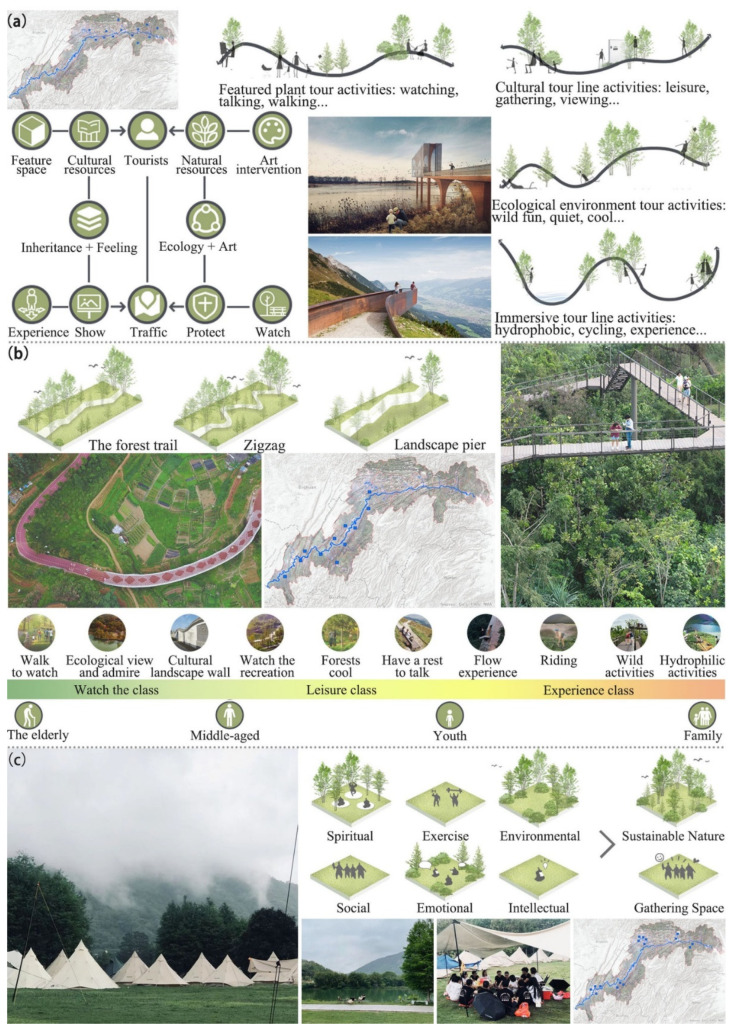
Landscape planning of landscape viewing platforms (**a**), immersive hiking routes (**b**), and gathering spaces (**c**) to attract more visitors and increase the cultural services of the TGRA.

First, we suggest building landscape-viewing platforms to improve the accessibility of the natural landscape (Figure 5a). Comprehensively integrating the cultural heritage landscape genes of the TGRA into these viewing platforms could help to showcase the region in all aspects. Meanwhile, we suggest construction of a unique, digital cultural landscape website of the reservoir area to share these natural landscapes. The website could contribute to displaying photography works; therefore, these viewing platforms are vital photography spots to help tourists find the best viewing position of the reservoir scenery and attract more visitors to go there. Four routes were designed to connect these viewing platforms: (1) a cultural route that connects with cultural heritage and could provide the functions of relaxation, gathering, and appreciation; (2) an eco-environment route that links with mountain and water landscape and is characterized by wildness, quietness, and coolness; (3) a feature vegetation route characterized by appreciation, conversation, and walking; and (4) an immersion route that ties in with cycling and experiencing activities. This landscape-planning scheme increases the diversity of motivations for travel, thus increasing the number of tourism visits to the TGRA. In addition, the setting of viewing platforms enhances the sense of participation, interaction, and benign bonding with the TGRA, which makes visitors have a better tourism experience.

Secondly, constructing an immersive hiking route links the mountain landscapes and cultural resources through the topographical combing of the reservoir landscape to strengthen the natural context (Figure 5b). These routes include forest trails, zigzag trails, and landscape bridges. We divided them into three major types of activities: ornamental, recreational, and experiential. These routes are characterized by strolling and viewing, ecological viewing, recreation and conversation, immersion experience, cycling activities, wildlife activities, and so on. Meanwhile, these routes are suitable for various groups, such as the elderly, middle-aged, youth, and families. In this landscape-planning scheme, we simultaneously provide a clear route and enough space for free exploration to satisfy visitors’ desire for adventure. Additionally, the layout of leisure areas should be interwoven with cycling and other off-road activities to mobilize the interest and enthusiasm of youth groups. The tourist route is designed with the starting point of experiencing the reservoir culture in many aspects so that it can generate a profound immersion experience and enhance the satisfaction of visitors.

Third, various gathering spaces, such as sports space, meditation space, and social space, in accordance with the natural, could be designed in the existing ecological environment of the reservoir area (Figure 5c). A private and peaceful outdoor space can also be set up among the trees to give people a sense of retreat. A wide range of interactive activities will meet the needs of different visitors in terms of spirituality, exercise, socialization, and emotions, encouraging them to get in touch with the ecosystem and create a memorable journey. The three zones not only provide visitors with different experiences but also encourage them to enter the site deeply and establish a profound connection with nature. It can also attract more tourists to the TGRA, which will promote the construction and development of tourism and strengthen the cultural services in the reservoir area.

### 4.5. Our Limitations and Uncertainties

In the Millennium Ecosystem Assessment framework, cultural services are an important component of ecosystem services. However, how to quantify the cultural services is the most difficult point in the current ecosystem service assessment and trade-off [17,18]. The InVEST model provides a spatial assessment with the number of visitors as a predictor variable and estimates the contribution of natural and cultural attributes to recreation services through a simple linear regression [35,36]. Recreation should have a non-linear relationship with natural and cultural attributes. However, the non-linear relationships are numerous in variety, and the complex non-linear regression equations are difficult to fit and interpret. The InVEST model attempts to analyze the response of cultural services represented by recreation to the natural ecological environment and human social environment using the simplest multiple linear regression. Although this is an inherent drawback of the model, the required data of this model are simple and effective in assessing the spatial characteristics of the recreation service [36]. We simulated the recreation service in the TGRA through the model for four periods, which helps to recognize and evaluate the ecosystem cultural services. Although there are some uncertainties in the assessment results, our study shifted the static ecosystem service assessment to an analysis of recreation service dynamics. On the contrary, it reflects the dynamic characteristics through multiple periods to reveal the spatial and temporal continuity of the recreation service in the TGRA. It also provides a detailed scientific basis for ecosystem cultural services regulation and governmental decision making.

## 5. Conclusions

Research on ecosystem services has been dominated by the natural sciences and economics, but cultural services research requires social science and humanistic perspectives to understand the concepts and practices of a place. As few studies have assessed the cultural service of the TGRA in previous papers, the impact of culture services on human activities at the regional scale are still unclear. In this study, we used the InVEST model to construct a multiple linear regression equation with an explanation rate of 0.75 to evaluate cultural services represented by recreation. Our result demonstrated that the proxy of recreation service at low elevation and gentle slope is higher than that at high elevation and steep slope, which indicated that the recreation-service supply capacity of the humanistic landscape is larger than that of the natural landscape. It also confirmed the incompleteness of infrastructure development and construction in these areas with higher altitudes and steeper slopes. In addition, the recreation-service proxy of the non-vegetation landscape is far higher than that of the vegetation landscape. However, ecological protection and urban construction in the reservoir area both could enhance the potential ability of the recreation service by increasing the potential recreation service of the natural landscape and strengthening the infrastructure development and construction, respectively. Therefore, we suggest planning the landscape and designing scenic forests for promoting the construction and development of tourism. There is a need to strengthen the construction of infrastructure in the high-altitude areas with beautiful natural landscapes and enhance the accessibility of scenic areas to attract tourists and upgrade the recreation service in the TGRA.

## Figures and Tables

**Table 1:** Index system for assessing recreation service.

Variable Category	Variable	Data Sources
Natural and environmental factors	Annual mean temperature (x1, °C)	Data Center for Resources and Environmental Sciences, Chinese Academy of Sciences (http://www.resdc.cn, accessed on 13 November 2018).
	Annual total precipitation (x2, mm)	
	Elevation (x3, m)	DEM (Digital Elevation Model) derived from ASTER Global Digital Elevation Model V002 (http://www.gscloud.cn/, accessed on 13 November 2018).
	Slope (x4, °)	Derived from DEM data.
	Soil organic carbon (x5, g/kg)	Derived from Huang et al. [25].
	Forest coverage (x6, %)	Derived from land use maps in 2000, 2005, 2010, and 2015 [34].
	Proportion of cropland area (x7, %)	
	Proportion of built-up area (x8, %)	
	Proportion of water area (x9, %)	
Socio-economic factors	Density of tourist attractions (x10, numbers/km^2^)	Points of tourist attractions were acquired from Baidu maps.
	Road density (x11, km/km^2^)	National Geomatics Center of China (NGCC) (http://ngcc.sbsm.gov.cn/, accessed on 13 November 2018).
	Minimum distance to traffic station (x12, km)	Points of traffic stations were acquired from Baidu maps.
	Minimum distance to hotel (x13, km)	Points of hotels were acquired from Baidu maps.

**Table 2:** Results of estimated parameters of multiple regression analysis.

Independent Variable	Estimated Value	Standard Errors	*t*-Value	Significance
Intercept	−21.27	10.82	−1.97	*p* < 0.05
Density of tourist attractions (numbers /km^2^)	0.44	0.02	17.66	*p* < 0.01
Minimum distance to traffic station (km)	−0.04	0.03	−1.08	*p* < 0.01
Minimum distance to hotel (km)	0.55	0.07	1.97	*p* < 0.05
Road density (km/km^2^)	7.77	0.03	3.24	*p* < 0.05
Elevation (m)	−0.01	0.01	−0.67	*p* < 0.05
Forest coverage (%)	0.34	0.02	2.18	*p* < 0.05
Proportion of built-up area (%)	−0.65	0.04	−4.32	*p* < 0.05
Proportion of water area (%)	1.69	0.09	1.79	*p* < 0.01

## Supplementary Materials

The following are available online at https://www.mdpi.com/article/10.3390/ijerph18168356/s1. Figure S1: Linear trend analysis of the recreation-service proxy of the TGRA; Figure S2: Average (a) and the modeled slope (b) of the recreation-service proxy between 2000 and 2015 at the county scale; Figure S3: Heat map of the correlation matrix and hierarchical clustering for 20 counties in the TGRA according to the recreation service between 2000 and 2015; Figure S4: Temporal variations of the recreation-service proxy for different land-use-change types of the TGRA.

## References

[1] Costanza R., D’Arge R., De Groot R., Farber S., Grasso M., Hannon B., Limburg K., Naeem S., O’Neill R.V., Paruelo J. (1997). The value of the world’s ecosystem services and natural capital. Nature.

[2] De Groot R.S., Alkemade R., Braat L., Hein L., Willemen L. (2010). Challenges in integrating the concept of ecosystem services and values in landscape planning, management and decision making. Ecol. Complex..

[3] Nowak-Olejnik A., Mocior E., Hibner J., Tokarczyk N. (2020). Human perceptions of cultural ecosystem services of semi-natural grasslands: The influence of plant communities. Ecosyst. Serv..

[4] Dou Y., Zhen L., De Groot R., Du B., Yu X. (2017). Assessing the importance of cultural ecosystem services in urban areas of Beijing municipality. Ecosyst. Serv..

[5] Geoffrey G., Andrew M. (2011). The Benefits of Physical Activity Provided by Park and Recreation Services: The Scientific Evidence. Australas. Parks Leis..

[6] Dong L., Zhu W., Gao Y., Li S. (2014). Research progress in culture ecosystem services (CES) and its development trend. Acta Sci. Nat. Univ. Pekin..

[7] Mao Q.-Z., Huang G.-L., Wu J.-G. (2015). Urban ecosystem services: A review. Chin. J. Appl. Ecol..

[8] Potwarka L.R., Havitz M.E., Glover T.D. (2015). Relationships among producer exposure, producer bias, and citizenship orientations in the context of public recreation service delivery. Loisir Société Society Leisure.

[9] Dickinson D.C., Hobbs R. (2017). Cultural ecosystem services: Characteristics, challenges and lessons for urban green space research. Ecosyst. Serv..

[10] Dou Y., Zhen L., Yu X., Bakker M., Carsjens G.-J., Xue Z. (2019). Assessing the influences of ecological restoration on perceptions of cultural ecosystem services by residents of agricultural landscapes of western China. Sci. Total Environ..

[11] Peng J., Hu X., Zhao M., Liu Y., Tian L. (2017). Research progress on ecosystem service trade-offs: From cognition to decision-making. Acta Geogr. Sin..

[12] Cabana D., Ryfield F., Crowe T.P., Brannigan J. (2020). Evaluating and communicating cultural ecosystem services. Ecosyst. Serv..

[13] Schirpke U., Scolozzi R., Dean G., Haller A., Jäger H., Kister J., Kovács B., Sarmiento F.O., Sattler B., Schleyer C. (2020). Cultural ecosystem services in mountain regions: Conceptualising conflicts among users and limitations of use. Ecosyst. Serv..

[14] Casado-Arzuaga I., Onaindia M., De Madariaga I., Verburg P.H. (2014). Mapping recreation and aesthetic value of ecosystems in the Bilbao Metropolitan Greenbelt (northern Spain) to support landscape planning. Landsc. Ecol..

[15] Malinga R.H., Gordon L., Jewitt G., Lindborg R. (2015). Mapping ecosystem services across scales and continents—A review. Ecosyst. Serv..

[16] Yao J., He X., Chen W. (2018). The latest progress in ecosystem service flow research methods. Chin. J. Appl. Ecol..

[17] Egoh B., Drakou E., Dunbar M., Maes J., Willemen L. (2012). Indicators for Mapping Ecosystem Services: A Review.

[18] Schägner J.P., Brander L., Paracchini M.L., Maes J., Gollnow F., Bertzky B. (2018). Spatial dimensions of recreational ecosystem service values: A review of meta-analyses and a combination of meta-analytic value-transfer and GIS. Ecosyst. Serv..

[19] Scholte S.S., van Teeffelen A., Verburg P. (2015). Integrating socio-cultural perspectives into ecosystem service valuation: A review of concepts and methods. Ecol. Econ..

[20] Richards D.R., Tuncer B. (2018). Using image recognition to automate assessment of cultural ecosystem services from social media photographs. Ecosyst. Serv..

[21] Thiele J., Albert C., Hermes J., von Haaren C. (2020). Assessing and quantifying offered cultural ecosystem services of German river landscapes. Ecosyst. Serv..

[22] Kosanic A., Petzold J. (2020). A systematic review of cultural ecosystem services and human wellbeing. Ecosyst. Serv..

[23] Zoderer B.M., Tasser E., Erb K.-H., Stanghellini P.S.L., Tappeiner U. (2016). Identifying and mapping the tourists perception of cultural ecosystem services: A case study from an Alpine region. Land Use Policy.

[24] Rall E., Bieling C., Zytynska S., Haase D. (2017). Exploring city-wide patterns of cultural ecosystem service perceptions and use. Ecol. Indic..

[25] Shoyama K., Yamagata Y. (2016). Local perception of ecosystem service bundles in the Kushiro watershed, Northern Japan—Application of a public participation GIS tool. Ecosyst. Serv..

[26] Zhang L. (2000). The exploration of layout strategy in tourism industry in the Yangtze Three Gorges Reservior. Econ. Geogr..

[27] Sun Z. (2001). Highlight the characteristics to create a boutique, strive to create a national garden city. J. Chin. Landsc. Archit..

[28] Tu J., Luo S., Yang Y., Qin P., Qi P., Li Q. (2021). Spatiotemporal Evolution and the Influencing Factors of Tourism-Based Social-Ecological System Vulnerability in the Three Gorges Reservoir Area, China. Sustainability.

[29] Cao H., Cheng C. (2014). Identification of the Non-Optimal Tourism Regions in the Three Gorges Area and Its Development Approach. Econ. Geogr..

[30] Huang C., Huang X., Peng C., Zhou Z., Teng M., Wang P. (2019). Land use/cover change in the Three Gorges Reservoir area, China: Reconciling the land use conflicts between development and protection. Catena.

[31] Liao Q., Wang Z., Huang C. (2020). Green Infrastructure Offset the Negative Ecological Effects of Urbanization and Storing Water in the Three Gorges Reservoir Area, China. Int. J. Environ. Res. Public Health.

[32] Xiang X. (2004). New Changes and Countermeasures in Tourism in Three Gorges of Yangtze River after Storing Water. J. Southwest China Norm. Univ. Philos. Soc. Sci. Ed..

[33] Huang C., Zhou Z., Teng M., Wu C., Wang P. (2020). Effects of climate, land use and land cover changes on soil loss in the Three Gorges Reservoir area, China. Geogr. Sustain..

[34] Yang G., Zhang F. Research on Spatiotemporal Differentiation and Influencing Factors of Tourism Development in Three Gorges Reservoir Area. Hubei Zhongke Geology and Environment Technology Institute. Proceedings of the 7th Academic Conference of Geology Resource Management and Sustainable Development, Hubei Zhongke Geology and Environment Technology Institute.

[35] Sharp R., Tallis H., Ricketts T., Guerry A., Wood S., Chaplin-Kramer R., Nelson E., Ennaanay D., Wolny S., Olwero N. (2014). InVEST User’s Guide.

[36] Tang Y., Zhu W., Zhang H., Song Y. (2015). A review on principle and application of the InVEST model. Ecol. Sci..

[37] Ruiz-Frau A., Ospina-Alvarez A., Villasante S., Pita P., Maya-Jariego I., de Juan S. (2020). Using graph theory and social media data to assess cultural ecosystem services in coastal areas: Method development and application. Ecosyst. Serv..

[38] Wu J., Zhong X., Peng J., Qin W. (2015). Function classification of ecological land in a small area based on ecosystem service bundles: A case study in Liangjiang New Area, China. Acta Ecol. Sin..

[39] Huang C.B., Teng M.J., Zeng L.X., Zhou Z.X., Xiao W.F., Zhu J.H., Wang P.C. (2018). Long-term changes of land use/cover in the Three Gorges Reservoir Area of the Yangtze River, China. Chin. J. Appl. Ecol..

[40] Wen Z., Wu S., Chen J., Lü M. (2017). NDVI indicated long-term interannual changes in vegetation activities and their responses to climatic and anthropogenic factors in the Three Gorges Reservoir Region, China. Sci. Total Environ..

[41] Hill M.O. (1974). Correspondence Analysis: A Neglected Multivariate Method. Appl. Stat..

[42] Feng Q., Zhao W., Fu B., Ding J., Wang S. (2017). Ecosystem service trade-offs and their influencing factors: A case study in the Loess Plateau of China. Sci. Total Environ..

[43] Sinclair M., Ghermandi A., Sheela A.M. (2018). A crowdsourced valuation of recreational ecosystem services using social media data: An application to a tropical wetland in India. Sci. Total Environ..

[44] Ferraro P.J., Hanauer M.M., Miteva D., Nelson J.L., Pattanayak S.K., Nolte C., Sims K. (2015). Estimating the impacts of conservation on ecosystem services and poverty by integrating modeling and evaluation. Proc. Natl. Acad. Sci. USA.

[45] Heagney E., Rose J., Ardeshiri A., Kovač M. (2018). Optimising recreation services from protected areas—Understanding the role of natural values, built infrastructure and contextual factors. Ecosyst. Serv..

[46] Richards D., Law A., Tan C., Shaikh S., Carrasco L., Jaung W., Oh R. (2020). Rapid urbanisation in Singapore causes a shift from local provisioning and regulating to cultural ecosystem services use. Ecosyst. Serv..

[47] Meng S., Huang Q., Zhang L., He C., Inostroza L., Bai Y., Yin D. (2020). Matches and mismatches between the supply of and demand for cultural ecosystem services in rapidly urbanizing watersheds: A case study in the Guanting Reservoir basin, China. Ecosyst. Serv..

[48] Prete C., Cozzi M., Viccaro M., Sijtsma F., Romano S. Social hotspots mapping: A participatory approach for identifying cultural ecosystem services of forests and semi-natural areas. Proceedings of the 8th AIEAA Conference.

[49] Jůza R., Jarský V., Riedl M., Zahradník D., Šišák L. (2020). Possibilities for Harmonisation between Recreation Services and Their Production within the Forest Sector—A Case Study of Municipal Forest Enterprise Hradec Králové (CZ). Forests.

