# Supplementary material for: Landscape Dynamics Improved Recreation Service of the Three Gorges Reservoir Area, China
Source: Int J Environ Res Public Health. 2021 Aug 6;18(16):8356. doi: 10.3390/ijerph18168356 (PMC8392286; doi:10.3390/ijerph18168356)
Supplement: Supplementary file 1 [file ijerph-18-08356-s001.zip › ijerph-1292186-supplementary.pdf]

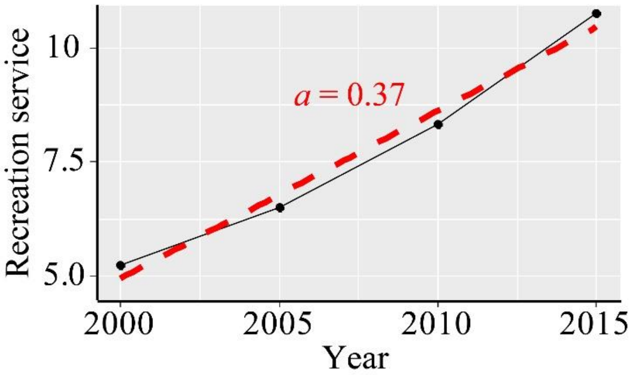

Figure S1. Linear trend analysis of the recreation service proxy of the TGRA. Note: In order to quantify the dynamic of recreation service, we used a least-square linear regression model to fit the recreation proxy. The changing trend is described by the modeled slope which is a in the figure. The black broken line is the estimated results, and the red dotted line is the trend line of the recreation.

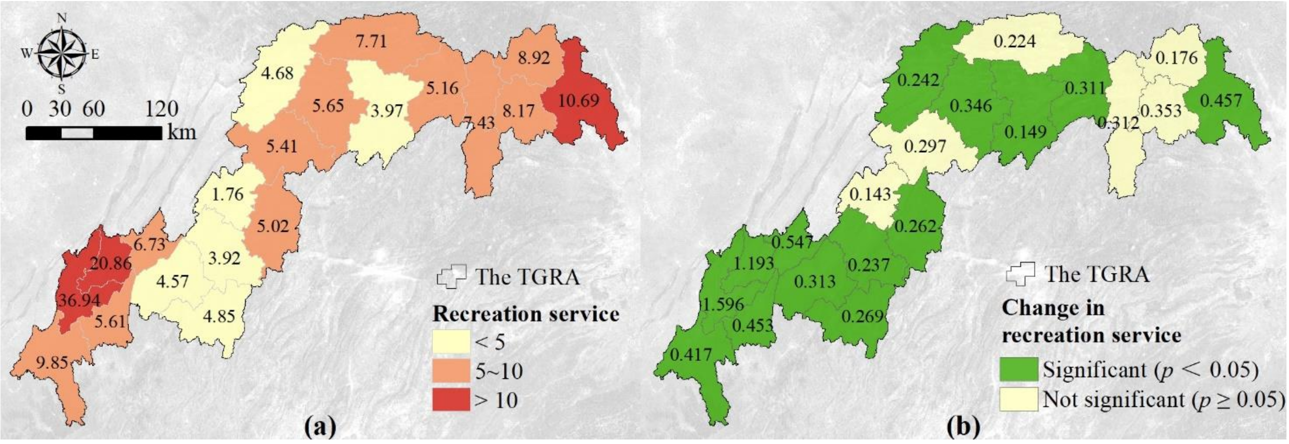

Figure S2. Average **(a)** and the modeled slope **(b)** of the recreation service proxy between 2000 and 2015 at the county scale.

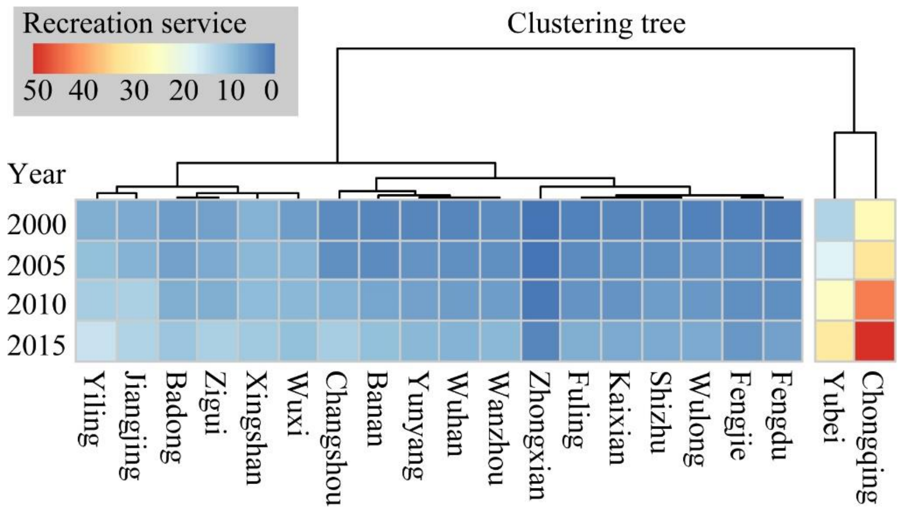

Figure S3. Heat map of the correlation matrix and hierarchical clustering for 20 counties in the TGRA according to the recreation service between 2000 and 2015.

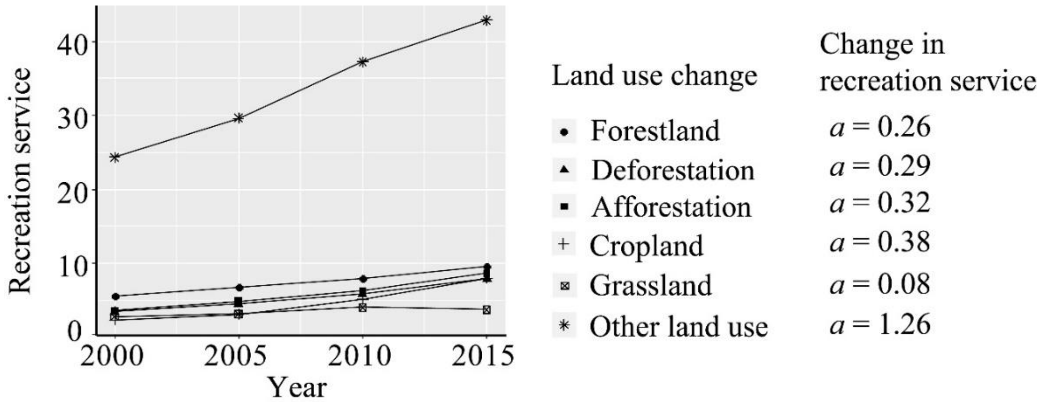

Figure S4. Temporal variations of the recreation service proxy for different land use change types of the TGRA. Note: We used a least-square linear regression model to fit the recreation. The changing trend is described by the modeled slope which is a in the figure.
